# Glucagon-like peptide-1 receptor agonists for major neurocognitive disorders

**DOI:** 10.1136/jnnp-2024-335593

**Published:** 2025-04-10

**Authors:** Riccardo De Giorgi, Ana Ghenciulescu, Courtney Yotter, Maxime Taquet, Ivan Koychev

**Affiliations:** 1 Department of Psychiatry, University of Oxford, Oxford, UK; 2 Oxford Health NHS Foundation Trust, Oxford, UK; 3 Division of Brain Sciences, Imperial College London, London, UK

**Keywords:** DEMENTIA, PARKINSON'S DISEASE, ALZHEIMER'S DISEASE

## Abstract

Disease-modifying treatments for major neurocognitive disorders, including Alzheimer’s disease, Parkinson’s disease and other cognitive deficits, are among the main unmet needs in modern medicine. Glucagon-like peptide-1 receptor agonists (GLP-1RAs), currently licensed for the treatment of type 2 diabetes mellitus and obesity, offer a novel, multilayered mechanism for intervention in neurodegeneration through intermediate, aetiology-agnostic pathways, likely involving metabolic, inflammatory and several other relevant neurobiological processes. In vitro and animal studies have revealed promising signals of neuroprotection, with preliminary supportive evidence emerging from recent pharmacoepidemiological investigations and clinical trials. In this article, we comprehensively review studies that investigate the impact of GLP-1RAs on the various aetiologies of cognitive impairment and dementia syndromes. Focusing on evidence from human studies, we highlight how brain energy homeostasis, neurogenesis, synaptic functioning, neuroinflammation and other cellular stress responses, pathological protein aggregates, proteostasis, cerebrovascular system and blood-brain barrier dynamics may underlie GLP-1RA putative neuroprotective effects. We then report and appraise evidence from clinical studies, including observational investigations, clinical trials and pooled analyses. Finally, we discuss current challenges and perspectives ahead for research and clinical implementation of GLP-1RAs for the care of people with major neurocognitive disorders, including their individual brain penetrance potential, the need for response biomarkers and disease stage-based indications, their possible non-specific effects on brain health, their profile in terms of adverse events and other unwanted effects, the lack of long-term data for efficacy and safety, and issues surrounding cost and availability of treatment.

## Introduction

 Major neurocognitive disorders such as Alzheimer’s disease (AD), vascular dementia (VaD) and Parkinson’s disease/Lewy body dementia (PD/LBD) are a major global health challenge.^1^ It is estimated that over 55 million people live with dementia globally, a number projected to reach more than 150 million by 2050.^2^ These diseases significantly impact patients and carers’ quality of life, while also placing a considerable burden on healthcare systems.^3^ Despite ongoing advances in understanding their physiopathology, effective treatments—especially disease-modifying ones—remain limited.^4^ In this context, glucagon-like peptide-1 receptor agonists (GLP-1RAs) are being proposed as potential drugs for managing cognitive disorders owing to their putative ability to affect neurobiological and metabolic pathways implicated in neurodegeneration.^5^

GLP-1RAs are a class of medications currently including exenatide, lixisenatide, dulaglutide, liraglutide, semaglutide and tirzepatide (a dual GLP-1/gastric inhibitory polypeptide (GIP) receptor agonist), initially developed for type 2 diabetes mellitus (T2DM).^6^ They mimic the action of endogenous GLP-1, which is secreted in response to nutrient intake and plays a key role in glucose homeostasis—hence the name ‘incretin mimetics’. GLP-1 binds to GLP-1 receptors distributed across different body systems, thereby enhancing insulin secretion, inhibiting glucagon release, slowing gastric emptying and reducing appetite. Intriguingly, GLP-1 and its receptor are widely expressed in regions of the central nervous system (CNS) associated with memory and learning such as the hippocampus and other higher cortical structures,^7^ as well as on neurons of the peripheral nervous system and the gut-brain axis.^8^ This distribution suggests a direct influence of the GLP-1 system on neural function, making GLP-1RAs promising candidates for addressing cognitive disorders.^9 10^ Their neuroprotective effects had already been postulated three decades ago when it was found that a component of Gila monster’s venom, the peptide exendin-4, could be synthesised in a drug called exenatide for use in diabetes, dementias and other chronic diseases common to older age.^11 12^

Some GLP-1RAs (ie, semaglutide, tirzepatide) have undergone a rapid expansion of their use beyond T2DM, including their approval for weight loss, the reduction of cardiovascular and renal morbidity and mortality,^13 14^ and more recently sleep apnoea.^15^ As they appear to modulate directly the central reward system (ie, ventral tegmental area, nucleus accumbens, hypothalamus, amygdala and others), these agents are also being tested in people with alcohol and other substance use disorders.^16^ However, the most significant body of both preclinical and clinical evidence is available for their repurposing in neurodegenerative disorders, advising that GLP-1RAs may enhance neuronal survival and delay disease progression.^17^ This review article explored the role of GLP-1RAs in treating cognitive disorders based on the most recent mechanistic and clinical findings, highlighting challenges and perspectives for their further development and potential healthcare applications.

## Mechanisms of neuroprotection

Evidence from genetic^18 19^ and proteomic^20 21^ analyses in humans suggests the involvement of the brain GLP-1/GIP (ie, incretin) system in neurodegeneration. Genetic variability in the receptors for GLP-1 (GLP-1R rs10305420, GLP-1R rs6923761) and GIP (GIPR rs1800437) has been associated with increased odds of AD and PD while correlating with elevated disease biomarkers (ie, amyloid-beta (Aβ) peptide 42, tau proteins clustered in neurofibrillary tangles (NFT)) measured in the cerebrospinal fluid (CSF).^19^ A Mendelian randomisation study from the UK Biobank data found an association between genetic variants coding for GLP-1RAs and a lower risk of AD—although these results must be interpreted cautiously as the study failed to show the expected associations with weight loss used as a positive control.^18^ Low levels of GLP-1 in serum were suggestive of higher odds of mild cognitive impairment (MCI) in a small sample of 106 patients with T2DM.^21^ More recently, a major study on the effects of the GLP-1RA semaglutide on the circulating proteome of some 2000 participants with overweight or obesity plus or minus diabetes from two phase III trials (STEP 1 and STEP 2) has identified two significantly regulated proteins, namely tenascin-C and progranulin, that are implicated in AD neuropathology.^20^

**Figure 1:**
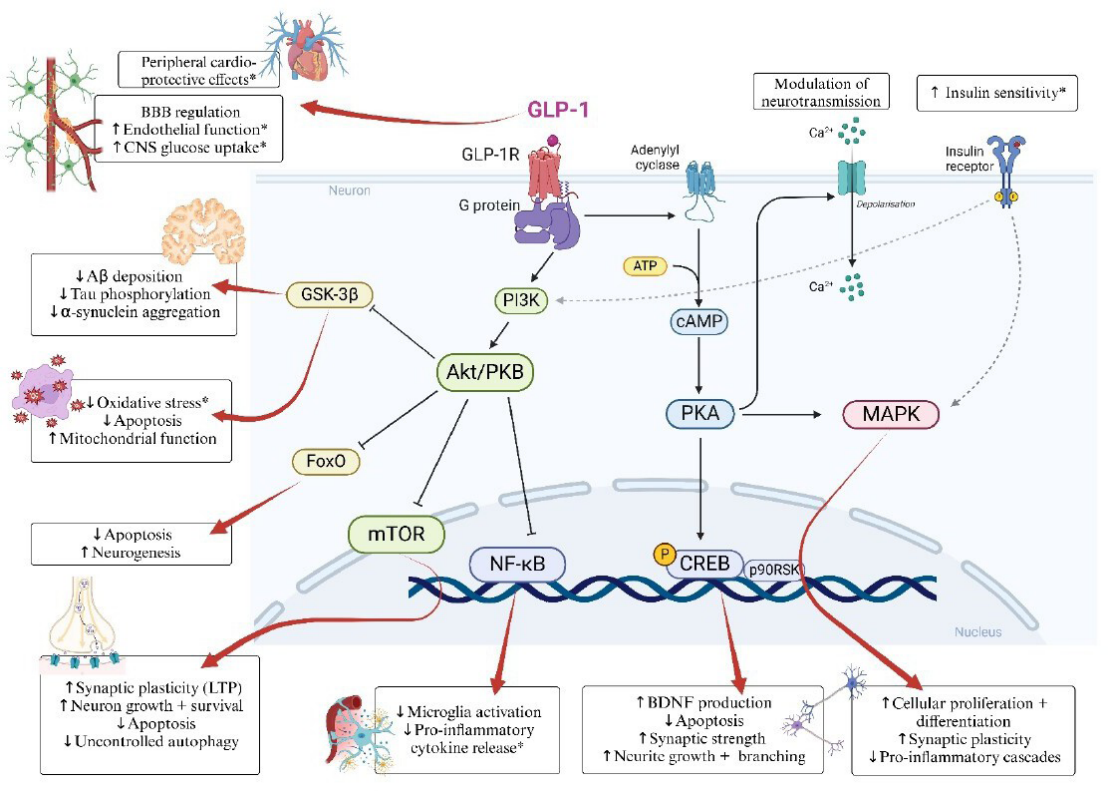
Molecular pathways underlying the putative neuroprotective effects of glucagon-like peptide-1 receptor agonists (GLP-1RAs). *Findings confirmed in human studies. ↑ denotes increase/enhance; ↓ denotes decrease/inhibit. Aβ, amyloid-beta; Akt/PKB, protein kinase B; BBB, blood-brain barrier; BDNF, brain-derived neurotrophic factor; cAMP, cyclic adenosine monophosphate; CNS, central nervous system; CREB, cAMP response element-binding protein; FOXO, forkhead box protein O; GSK-3β, glycogen synthase kinase 3 beta; LTP, long-term potentiation; MAPK, mitogen-associated protein kinase; mTOR, mammalian target of rapamycin; NF-κB, nuclear factor kappa B; p90RSK, p90 ribosomal kinase; PI3K, phosphoinositide 3-kinase; PKA, protein kinase A.^122^

From a mechanistic perspective, however, most evidence regarding the putative neuroprotective activity of GLP-1RAs stems from preclinical, that is, in vitro or animal models.^10 17^ In summary, beyond peripheral glycaemic control, GLP-1RAs display broad actions against neurodegeneration and ageing processes while being capable of enhancing cognitive abilities. Observed cross-domain effects involve: brain energy homeostasis; neurogenesis and synaptic functioning; neuroinflammation and other cellular stress responses; pathological protein aggregates and proteostasis; and cerebrovascular system and blood-brain barrier (BBB) dynamics—see figure 1. Some cognitive effects may also be the indirect effect rather than the direct result of the direct entry of GLP-1RAs in the CNS, but rather be indirectly related to the functions that these medications express in the periphery,^9^ possibly across immune, endocrine-metabolic and gut-brain axis^17^—see also the ‘Challenges and perspectives for research and clinical practice section and the Brain penetrance section’.

### Brain energy homeostasis

Impaired insulin signalling in the brain has been strongly associated with AD and other dementias.^22^ GLP-1RAs that enter the CNS have been proposed to locally improve insulin sensitivity, thus restoring energy balance within neural circuits and reducing hyperglycaemic toxicity as observed in preclinical models.^9 10 23^

In humans, evidence for this mechanism was obtained in a 6-month randomised placebo-controlled trial of 38 people with AD, where liraglutide prevented the decline of glucose metabolism^24^ and restored glucose transport at the BBB^25^ as measured with [^18^F]Fluorodeoxyglucose positron emission tomography (PET)—see also ref 26. In PD, a randomised placebo-controlled trial of exenatide in 60 patients followed up for 48 weeks similarly showed target engagement of brain insulin and protein kinase B (Akt)/phosphorylated mechanistic target of rapamycin signalling pathways correlating with disease progression.^27^

### Brain structure and connectivity

Altered neuronal homeostasis and connectivity are commonly observed in neurodegeneration^28^—preclinical evidence indicates that the incretin system and GLP-1RAs are implicated in these processes through pathways like Akt/cAMP response element-binding protein/brain-derived neurotrophic factor, other neurotrophins such as glial cell line-derived neurotrophic factor and long-term potentiation.^9 10 29^

Findings from several studies conducted in humans are in keeping with the above. A 12-month randomised placebo-controlled trial of liraglutide in 204 people with mild to moderate AD found that patients treated with liraglutide had a lower rate of temporal lobe and whole cortical volume loss on MRI.^30^ This contrasts with an earlier, considerably smaller (18 participants) trial of another GLP-1RA, exenatide, which could not show any difference in MRI cortical thickness and volume at 18 months.^31^ In addition, a recent meta-analysis of eight trials found that exenatide increases functional connectivity in the hypothalamus, nucleus tractus solitarius and thalamus, and that liraglutide increases connectivity of the hippocampus, while other GLP-1RAs such as dulaglutide decrease functional connectivity in the hypothalamus, orbitofrontal cortex (OFC) and amygdala.^32^ Overall, endogenous and pharmacological GLP-1RAs appear to modulate connectivity within several functional networks including the dorsal default mode network, visuospatial network, right frontal parietal network and the salience network.^32^ Two studies, not included in the meta-analysis, of 36 and 50 patients with T2DM, respectively, confirmed the effect of liraglutide on increasing hippocampal activation^33^ and further identified increased activity within the dorsolateral prefrontal cortex (DLPFC) and OFC.^34^

### Neuroinflammation and other cellular stress responses

Maladaptive neuroinflammatory phenomena are recognised as critical for the development of neurodegenerative disorders,^35^ as are other reactive oxygen species, mitochondrial functioning and endoplasmic reticulum stress.^36 37^ In vitro and animal studies show that GLP-1RAs have potent anti-inflammatory properties in the brain, both directly and indirectly, as they can moderate proinflammatory cytokine release, nuclear factor kappa B signalling and microglial activation, while also regulating oxidative and unfolded protein stress responses and mitophagy.^7 9 10 38^

A key analysis of the Exenatide Study of Cardiovascular Event Lowering (EXSCEL) trial (13 752 people with T2DM) found that 1 year of exenatide use, compared with placebo, reduced several inflammatory proteins conventionally associated with AD, including ficolin-2, plasminogen activator inhibitor 1 (PAI-1), soluble vascular cell adhesion protein 1 and a cytokine-cytokine cluster.^39^ Furthermore, a small trial in 60 participants assigned to either sitagliptin or liraglutide showed significantly decreased levels of serum inflammatory markers (C reactive protein, tumour necrosis factor‐alpha and interleukin-6) at 6 months in both groups, but more so in those receiving sitagliptin.^40^

Antioxidant effects, assessed as Trolox equivalent of antioxidant capacity of dulaglutide against placebo, were seen in a small sample of 25 subjects with multiple sclerosis.^41^

### Pathological protein aggregates and proteostasis

A hallmark of neurodegenerative diseases is the presence of abnormal extracellular (eg, Aβ for AD) and/or intracellular (eg, tau/NFT for AD, α-synuclein for PD) deposits.^42^ A substantial amount of preclinical data support GLP-1RA interaction with neurodegeneration proteinopathies,^9 10 43 44^ including a meta-analysis of 26 animal studies reporting decreasing Aβ sheets and phosphorylated tau accumulation following GLP-1RA use, along with improved learning and memory measures.^45^ Notably, other studies have not identified such positive outcomes in mice models of AD^46^ and PD.^47^

Literature on the effect of GLP-1RAs on protein aggregates and proteostasis in humans is scarce and inconsistent: while some tentative evidence from randomised placebo-controlled trials suggests that liraglutide reduces Aβ load in patients with MCI^40^ or AD^24 31^ after 6–18 months of treatment, larger analyses have not observed any effect on these AD biomarkers.^26^ To try and resolve these inconsistencies, our team is conducting a randomised placebo-controlled trial (Impact of Semaglutide in Amyloid Positivity) of oral semaglutide that will recruit 88 community-dwelling UK adults aged 55+ years with amyloid positivity on PET or CSF and no or MCI at baseline, with a view to assessing changes in tau and neuroinflammatory PET signal at 1 year.^48^

### Cerebrovascular system and BBB dynamics

Disrupted integrity of cerebral vessels and adequate BBB functioning contribute to a range of cognitive disorders including VaD and mixed dementia presentations.^49^ Some preliminary evidence suggests that GLP-1RAs may improve neurovascular and endothelial health.^50^

We found one cross-sectional investigation of 154 elderly patients with T2DM showing that treatment with GLP-1RAs plus metformin, compared with metformin alone, was associated with higher circulating levels of endothelial progenitor cells and improved cognition.^51^ Another study in 25 people with multiple sclerosis reported that dulaglutide was better than placebo at preserving endothelial function expressed as reperfusion hyperaemia index.^41^ Importantly, the ability for GLP-1RAs to regulate BBB dynamics would also affect their ability to enter the CNS (see the Challenges and perspectives for research and clinical practice section).

## Clinical studies in major neurocognitive disorders

The role of GLP-1RAs in dementia and other neurodegenerative diseases has long been suggested,^11 12^ but clinical evidence for such uses has only recently become available with a rapid growth over the past few years. In this section, we will consider studies focused on dementia, including AD; PD and LBD; and other cognitive deficits.

### Dementia, including AD

Clinical studies relevant to this section are summarised in table 1.

**Table 1:** Clinical studies of GLP-1RAs for dementia, including Alzheimer’s disease

	Population	Exposure	Comparator	Follow-up	Outcomes
Pooled and combined analyses					
Kuate Defo *et al*^73^	140 000 adults, T2DM (1 pooled analysis, 2 observational studies)	GLP-1RAs	Non-users of GLP-1RAs	7.2–14 years	Reduced risk of dementia with GLP-1RAs.
Li *et al*^71^	195 983 adults, T2DM (5 observational studies)	GLP-1RAs	Non-users of GLP-1RAs	5–22 years	GLP-1RAs ranked second after SGLT‐2i for reducing risk of all-cause dementia. Lower risk of AD with GLP-1RAs.
	37 176 adults, T2DM (4 RCTs)		Placebo	1.2–5.4 years	Risk of dementia comparable among all antidiabetics.
Liang *et al*^26^	144 adults, AD or at risk of AD (4 RCTs)	Exenatide, liraglutide	Placebo	3–18 months	No improvement in cognitive measures or AD biomarkers.
Nørgaard *et al*^69^	15 820 adults, T2DM (3 RCTs)	Liraglutide, semaglutide	Placebo	7.4 years	Lower rates of dementia with GLP-1RAs.
Tang *et al* ^70^	210 521 adults, T2DM (1 pooled analysis, 3 observational studies)	GLP-1RAs	Non-users of GLP-1RAs	3.6–7.4 years	Reduced risk of all-cause dementia with GLP-1RAs.
Tian *et al* ^72^	149 560 adults, T2DM (1 RCT, 3 observational studies)	GLP-1RAs	Non-users of GLP-1RA	4–7.2 years	GLP-1RAs ranked second after SGLT‐2i for reducing dementia risk.

Clinical trials					
Edison *et al*^30^	204 adults, AD	Liraglutide	Placebo	1 year	Improved ADAS-EXEC scores and higher temporal lobe and whole cortical MRI volumes with liraglutide.
Gejl *et al*^24^	38 adults, AD	Liraglutide	Placebo	6 months	No changes in cognitive scores (the study was underpowered).
Mullins *et al*^31^	21 adults, early AD	Exenatide	Placebo	1.5 years	No changes in cognitive scores, MRI measures or biomarkers (the study was underpowered, terminated early).

Observational studies					
Akimoto *et al*^55^	66 085 older adults, T2DM (1250 concomitant AD)	Dulaglutide, exenatide, liraglutide	Metformin	14 years	Significantly lower risk of AD with GLP-1RA monotherapy.
Bohlken *et al*^52^	16 552 adults, T2DM	Patients with dementia on GLP-1RAs	Patients without dementia on GLP-1RAs	5 years	No significant difference in risk of dementia with GLP-1RAs.
De Giorgi *et al*^57^	38 412–46 772 adults, T2DM	Semaglutide	Empagliflozin, glipizide, sitagliptin	1–2 years	Reduced risk of dementia with semaglutide compared with sitagliptin only.
Hong *et al* (2024)^64^	13 564 older adults, T2DM	Dulaglutide	Dapagliflozin, empagliflozin	5 years	No significant differences in the risk of dementia between those on GLP-1RAs and SGLT-2is.
Nassar (2025)^61^	854 201 adults, T2DM; 230 782 adults, obesity	GLP-1RAs	No active comparator	5 years	Significantly lower risk of all-cause dementia, AD and PD in users of GLP-1RAs with T2DM.
Nørgaard *et al*^69^	120 054 adults, T2DM	GLP-1RAs	Non-users of GLP-1RAs	7.4 years	Reduced risk of dementia with increasing exposure to GLP-1RAs.
Pai *et al*^63^	331 132 adults, T2DM	GLP-1RAs	SGLT-2is	Variable	Higher risk of dementia and all-cause mortality with GLP-1RAs compared with SGLT-2is.
Secnik *et al*^54^	133 318 adults, diabetes	Patients with dementia on GLP-1RAs	Patients without dementia on GLP-1RAs	14 years	Lower annual GLP-1RA dispensation in patients with dementia.
Siddeeque *et al*^62^	205 870 adults, obesity	GLP-1RAs	No active comparator	1 year	Significantly lower risk of AD, LBD and vascular dementia in users of any GLP-1RA.
Tang *et al* (2024)^59^	88 381 older adults, T2DM	GLP-1RAs	DPP-4is or sulfonylureas	4.3 years	Reduced risk of dementia in GLP-1RA users.
Wang et *al* (2024)^58^	1 094 761 adults, T2DM	Semaglutide	Other antidiabetic agents	3 years	Significantly lower risk of AD diagnosis with semaglutide (smallest effect size when compared with other GLP-1RAs).
Wium-Andersen *et al*^53^	58 095 adults, T2DM	GLP-1RAs	Non-users of GLP-1RAs	7.2 years	Lower odds of dementia with GLP-1RAs proportional to daily dose.
Xie *et al*^65^	215 970 adults, diabetes	GLP-1RAs	DPP-4is, SGLT-2is, sulfonylureas or usual care	3.7 years	Reduced risk of dementia and AD in GLP-1RA users compared with those continuing usual care.
Zamora *et al*^56^	29 260 adults on antidiabetics	GLP-1RAs	Non-users of GLP-1RAs	3 years	GLP-1RA use associated with lower odds of anti-AD drug prescriptions in 70–80 year-olds.
Zhou *et al*^60^	342 608 adults, T2DM	Exenatide	No active comparator	5 years	Lower incidence of AD with exenatide users >65 years old.

AD, Alzheimer’s disease; ADAS-EXEC, Alzheimer’s Disease Assessment Scale with Executive domains; DPP-4i, dipeptidyl peptidase-4 inhibitor; GLP-1RA, glucagon-like peptide-1 receptor agonist; LBD, Lewy body dementia; PD, Parkinson’s disease; RCT, randomised controlled trial; SGLT-2i, sodium-glucose co-transporter-2 inhibitor; T2DM, type 2 diabetes mellitus.

#### Observational studies

Most current evidence regarding the possible benefits of GLP-1RAs on the clinical risk of dementia comes from observational investigations.

Most studies did not differentiate between dementia subtypes. Earlier studies had a case–control design and examined associations between GLP-1RAs as a class and dementia risk in people with T2DM. One study detected no association between GLP-1RA use and dementia risk (OR 0.90; 95% CI 0.70, 1.15) over 5 years in 8276 patients with T2DM and dementia (1.7% on any GLP-1RAs) and 8276 patients with T2DM but without dementia (2.1% on any GLP-1RAs) across general practices in Germany between 2013 and 2017.^52^ Another study observed lower odds of dementia (OR 0.58; 95% CI 0.50, 0.67) with GLP-1RAs, which was proportional to daily dosage, in 11 619 cases of dementia and 46 476 controls from the Danish National Diabetes Register between 1995 and 2012 and followed up until 2018—notably, the same applied to all other antidiabetics (ie, metformin, dipeptidyl peptidase-4 inhibitors (DPP-4i), sodium-glucose co-transporter-2 inhibitors (SGLT-2i)) included in the analysis.^53^ Similarly, a registry-based study on 133 318 Swedish individuals with any diabetes (9.2% with dementia) between 2005 and 2018 found lower probability of GLP-1RA dispensations (HR 0.51; 95% CI 0.41, 0.63), and the same for DPP-4i and SGLT-2i.^54^

Two studies investigated the risk of AD specifically. A survey of the US Food and Drug Administration Adverse Event Reporting System over 66 085 patients aged 65+ years with T2DM (1250 with concomitant AD) showed lower odds of AD in people on exenatide compared with metformin (adjusted reporting OR (aROR) 0.22; 95% CI 0.11, 0.37), liraglutide compared with metformin (aROR 0.36; 95% CI 0.19, 0.62) and dulaglutide compared with metformin (aROR 0.39; 95% CI 0.17, 0.77).^55^ A cross-sectional investigation of 29 260 people on antidiabetics between 2018 and 2020 in Spain reported that GLP-1RA use was associated with lower anti-AD drug prescriptions in those aged 70–80 years (OR 0.57; 95% CI 0.45, 0.73).^56^

Compared with these analyses, cohort designs provide more robust estimates of risk; towards the end of 2024, six such studies were published. First, we found that patients with T2DM with semaglutide had lower hazards of dementia at 1 year after prescription when compared with the DPP-4i sitagliptin (n=46 772; HR 0.52; 95% CI 0.40, 0.68), and similar results when compared with the sulfonylurea glipizide (n=38 412; HR 0.63; 95% CI 0.46, 0.86)—though statistical significance was not reached for the latter following adjustment for multiple comparisons.^57^ Then, an independent group conducted an emulated target trial (ETT) over a 3-year follow-up on the same database in 1+ million patients with T2DM and found that semaglutide was associated with reduced risk of AD diagnosis specifically when compared with insulin (HR 0.33; 95% CI 0.20, 0.51) and other GLP-1RAs (HR 0.59; 95% CI 0.37, 0.95).^58^ Comparable findings in older adults (ie, 65+ years old) with T2DM followed between 2010 and 2020 were reported by another group using Swedish National Registers, showing a reduced risk of dementia for all GLP-1RA users (n=12 351) compared with propensity score-matched DPP-4i (HR 0.77; 95% CI 0.68, 0.88) and sulfonylurea (HR 0.69; 95% CI 0.60, 0.79) users,^59^ and also in AD in a separate study for exenatide which used claims data from a 20% random sample of Medicare beneficiaries with T2DM.^60^ Two further studies using TriNetX electronic health records (EHRs) but without an active comparator group found a beneficial association between GLP-1RAs and dementia risk in people with T2DM and obesity.^61 62^ On this same dataset, SGLT-2i was found to be slightly superior to GLP-1RAs for dementia risk (HR 0.92; 95% CI 0.87, 0.98) in another study of 331 132 patients who were also on metformin between 2012 and 2022.^63^ Conversely, no significant differences were observed in a smaller ETT of South Korean National Health Insurance records over 5 years comparing 1075 patients on the GLP-1RA dulaglutide against two SGLT-2is.^64^ Finally, a study of US Department of Veterans Affairs databases has used a discovery approach to systematically map an atlas of the associations of GLP-1RA use (n=215 970) against various comparators, revealing significantly lower associations with several cognitive disorders including AD.^65^

Taken together, these findings suggest that GLP-1RAs are associated with a lower risk of dementia, but differences among compounds within this class are possible. Nevertheless, variability in study designs and outcome measures complicates direct comparisons within the available data.

#### Clinical trials

Three early-phase randomised placebo-controlled trials of GLP-1RAs in people with a diagnosis of dementia have been published. Neither liraglutide administration over 6 months^24^ nor exenatide for 18 months^31^ led to any changes in cognitive scores in small samples (n=38 and 21, respectively) of patients with AD. A phase IIb multicentre trial in the UK is further evaluating the effects of liraglutide in mild to moderate AD at 12 months (ELAD, NCT01843075)^66^: a preliminary analysis in 204 patients showed better cognitive function measured on the Alzheimer’s Disease Assessment Scale-Executive Domain scale,^30^ and an update presented at the Alzheimer’s Association International Conference in 2024 revealed that this medication significantly reduced cognitive decline by 18% compared with placebo, even though this was a secondary outcome which should therefore be interpreted cautiously.^67^ A large phase III trial (evoke/evoke+) assessing the efficacy, safety and tolerability of oral semaglutide over 3 years, along with informative plasma and CSF biomarkers, in early-stage symptomatic AD is ongoing and expected to be completed in September 2025.^68^ While these results are pending, the paucity of evidence from randomised data in small samples of patients should be interpreted cautiously.

#### Pooled and combined analyses

A number of meta-analyses and pooled analyses of selected studies have been published. A prespecified pooled analysis of three randomised placebo-controlled trials along with a Danish registry-based cohort study including 15 820 and 120 054 patients with T2DM (ie, no baseline dementia), respectively, showed better dementia outcomes (HR 0.47; 95% CI 0.25, 0.86 at 4 years and HR 0.89; 95% CI 0.86, 0.93 at 7 years) for both liraglutide and semaglutide among GLP-1RAs.^69^ This study was included in another analysis including both randomised and some of the non-randomised data previously presented and reported a lower risk of all-cause dementia associated with any GLP-1RA.^70^ Two further network meta-analyses of selected publications^71 72^ ranked GLP-1RAs second for reducing the risk of dementia, including AD. A combined analysis of four clinical trials including both people with and without AD did not report any effect of GLP-1RAs on cognitive outcomes.^26^ Most recently, an umbrella review covering a selected portion of the available literature has argued that GLP-1RAs may be beneficial in dementia.^73^

All these combined analyses have several methodological issues (including unclear study selection process and inadequate assessment of clinical and methodological heterogeneity of included studies), preventing any robust recommendation to be made on the use of GLP-1RAs in dementia. Little data are available for dementia subtypes other than AD for which GLP-1RAs may present particular benefits (see the Challenges and perspectives for research and clinical practice section).

### PD and LBD

Clinical studies relevant to this section are summarised in table 2.

**Table 2:** Clinical studies of GLP-1RAs for Parkinson’s disease

	Population	Exposure	Comparator	Follow-up	Outcomes
Pooled and combined analyses					
de Albuquerque *et al*^84^	484 adults, PD (5 RCTs)	Exenatide, NLY01, liraglutide, lixisenatide	Placebo	9 months to 1 year	Significant improvements in motor and cognitive outcomes (MDS-UPDRS, MDRS2) with GLP-1RAs.

Clinical trials					
Aviles-Olmos *et al*^78^	45 adults, moderate PD	Exenatide	No active comparator	1 year	Improvement in cognitive and off-medication motor measures with exenatide (MDS-UPDRS).
Aviles-Olmos *et al*^79^	44 adults, moderate PD	Exenatide	No active comparator	2 years	Exenatide-treated patients had sustained improvements in motor symptoms (MDS-UPDRS) and cognition (MDRS2).
Athauda (2018)^80^	62 adults, moderate PD	Exenatide	Placebo	1 year	Exenatide improved off-medication motor symptoms (MDS-UPDRS), sustained 12 weeks after exposure.
Athauda *et al*^80^	62 adults, moderate PD	Exenatide	Placebo	1 year	Improvements in mood (NMSS, MDS-UPDRS) and emotional well-being (PDQ-39) with exenatide.
Meissner *et al*^82^	156 adults, early PD	Lixisenatide	Placebo	1 year	Treatment with lixisenatide reduced progression of motor disability (MDS-UPDRS part III).
McGarry *et al*^83^	255 adults, early PD	NLY01 (long-acting exenatide)	Placebo	9 months	No change in motor or non-motor features (MDS-UPDRS) with NLY01. May improve motor symptoms in younger patients.
Vijiaratnam *et al*^81^	194 adults, mild to moderate PD	Exenatide	Placebo	2 years	No improvements in motor or cognitive outcomes (MDS-UPDRS, NMSS, PDQ-39, MoCA, PHQ-9) with exenatide. No changes in striatal binding on DaT-SPECT.

Observational studies					
Brauer *et al*^75^	100 288 adults, T2DM	GLP-1RAs; DPP-4is	Other oral antidiabetics	3.3 years	Lower rate of PD diagnosis in patients using GLP-1RAs or DPP-4 compared with other antidiabetics.
De Giorgi *et al*^57^	38 412–46 772 adults, T2DM	Semaglutide	Empagliflozin, glipizide, sitagliptin	1–2 years	No associations between GLP-1RAs and PD risk.
Nassar (2025)^61^	854 201 adults, T2DM; 230 782 adults, obesity	GLP-1RAs	No active comparator	5 years	Significantly lower risk of PD in users of GLP-1RAs.
Rozani *et al*^76^	86 229 adults, T2DM	GLP-1RAs	Metformin	9 years	Significantly lower risk of PD with GLP-1RAs.
Siddeeque *et al*^62^	205 870 adults, obesity	GLP-1RAs	No active comparator	1 year	Significantly lower risk of PD with semaglutide only.
Sunnarborg *et al*^74^	9951 adults, diabetes	Patients with PD on GLP-1RAs	Patients without PD on GLP-1RAs	20 years	No association between GLP-1RAs and risk of PD (similar to other antidiabetics investigated).
Tang *et al* (2024)^77^	89 074 older adults, T2DM	GLP-1RAs	DPP-4is	5 years	Lower risk of PD in users of GLP-1RAs.

DaT-SPECT, dopamine transporter single-photon emission CT; DPP-4i, dipeptidyl peptidase-4 inhibitor; GLP-1RA, glucagon-like peptide-1 receptor agonist; MDRS2, Mattis Dementia Rating Scale; MDS-UPDRS, Movement Disorders Society Unified Parkinson's Disease Rating Scale; MoCA, Montreal Cognitive Assessment; NMSS, Non-Motor Symptom Scale; PD, Parkinson’s disease; PDQ-39, Parkinson's Disease Questionnaire; PHQ-9, Patient Health Questionnaire; RCT, randomised controlled trial; T2DM, type 2 diabetes mellitus.

#### Observational studies

Retrieved observational work relevant to GLP-1RAs and the risk of PD/LBD in people with T2DM has only been published within the last few years.

A case–control study of a nationwide Finnish register in patients with diabetes (2017 cases, 7934 controls) between 1999 and 2015 reported no association between any antidiabetics, including GLP-1RAs (OR 0.90; 95% CI 0.45, 1.80), and the odds of PD over 20 years.^74^

A larger (n=100 288), more robust cohort investigation of primary care data from the UK, The Health Improvement Network, between 2006 and 2019 across a 3-year follow-up observed a lower incidence rate ratio (0.38; 95% CI 0.17, 0.60) of PD for GLP-1RAs, along with DPP-4i, compared with other oral antidiabetics.^75^ Compared with metformin alone, GLP-1RA use was still less associated with diagnoses of PD (n=86 229; HR 0.54; 95% CI 0.39, 0.73) on Maccabi Healthcare Services data between 1999 and 2018 with a 9-year follow-up.^76^ Four studies surveyed US healthcare records. Of these, three were on the TriNetX US Collaborative Network, but two—which reported a potentially protective association between GLP-1RAs and PD/LBD diagnoses in T2DM and/or obesity—did not use an active comparator nor accounted for multiple comparisons,^61 62^ whereas one found no association between semaglutide and PD diagnosis in 38 412+ people with T2DM over 1+ years, compared with other individual antidiabetics at the same level of treatment (ie, the DPP-4i sitagliptin, the SGLT-2i empagliflozin and the sulfonylurea glipizide).^57^ Using US Medicare administrative data of older adults only between 2016 and 2020, however, GLP-1RA users were shown to be at lower risk of PD (HR 0.77; 95% CI 0.63, 0.95) than DPP-4i users.^77^ In other words, observational evidence for GLP-1RAs and PD/LBD risk remains conflicting and, at present, does not provide clear directions for research and clinical practice.

#### Clinical trials

Compared with the low number of trials of GLP-1RAs in people with an established diagnosis of dementia, it is interesting to notice that many of these studies have been accomplished—some published as early as in 2013 for PD/LBD. Most clinical trials investigated the older GLP-1RA exenatide. A UK-based proof-of-concept single-blind trial evaluated the progress of 45 patients with moderate PD randomly assigned to exenatide or control and saw better scores (mean difference (MD) 4.9; 95% CI 0.3, 9.4) for the intervention group at 12 months as assessed by the Movement Disorders Society Unified Parkinson’s Disease Rating Scale (MDS-UPDRS),^78^ which measures both motor and non-motor outcomes. Such improvement was still noticeable and indeed even more pronounced (MD 5.6; 95% CI 2.2, 9.0) after another 12 months without medication (ie, 24 months from baseline), and was also associated with a better score (MD 5.3; 95% CI 1.4, 9.3) on the Mattis Dementia Rating Scale.^79^ Two major studies were later published by the same UK research group who conducted an analogous randomised placebo-controlled trial, though double blind, in 62 patients with moderate PD on antiparkinsonian treatment and confirmed an effect favouring add-on exenatide (MD −3.5; 95% CI −6.7, –0.3) on the MDS-UPDRS^80^ as well as on several mood-related non-motor scores^80^ at 48–60 weeks, though intriguingly the latter seemed to fade off after a 12-week washout period from the medication. Contrastingly, their latest phase III multicentre UK trial for exenatide in 194 people with mild to moderate PD (exenatide-PD3) has only recently been completed and found no difference between drug and placebo over 2 years, suggesting that this medication may not have disease-modifying potential in this group of patients.^81^ The pharmacologically akin GLP-1RA lixisenatide has recently been investigated in a key phase II randomised placebo-controlled trial (LIXIPARK) involving 156 people with early PD in France: an improvement in motor symptoms only on the MDS-UPDRS part 3 was found at 12 months (MD 3.08; 95% CI 0.86, 5.30), along with significantly worse gastrointestinal side effects.^82^ Results have recently been made available for a novel GLP-1RA compound named NLY01—a brain-penetrant, pegylated, longer lasting version of exenatide; in a US-based randomised placebo-controlled trial over 255 participants with early untreated PD, 36 weeks of treatment with different medication doses did not lead to any appreciable difference (ie, small effect sizes, very large CIs) in motor and non-motor symptoms compared with placebo.^83^ We further retrieved a non-peer-reviewed preprint of another US phase II trial (NCT02953665) of liraglutide (n=42) versus placebo (n=21) in patients with PD that reported several better outcomes for the intervention group, but no update nor publication has been made available since 2022. On the whole, evidence from randomised data seems promising and should be suggestive of further research investment, but to the best of our knowledge, we could not find any further ongoing trials, especially for newer GLP-1RAs, in PD.

#### Pooled and combined analyses

Once again in contrast with the other sections, and despite the availability of several trials, only one meta-analysis is available for GLP-1RAs in PD—this appropriately included all trials above but for the most recent one, which was published after this meta-analysis.^81^ Across five clinical trials including 484 adults with PD at various disease stages, GLP-1RA administration (ie, exenatide, NLY01, liraglutide, lixisenatide) determined significant improvements in global symptoms (standardised mean difference (SMD) −3.43; 95% CI −6.48, –0.48), motor symptoms (SMD −2.52; 95% CI −4.02, –1.01 on medication and SMD −1.22; 95% CI −2.46, 0.22 off medication) and cognitive symptoms (SMD 1.32; 95% CI 0.16, 2.52) over 1 year.^84^ These findings are eloquent and suggest that further trials with longer follow-ups are needed to establish the disease-modifying potential of GLP-1RAs in a chronic degenerative condition such as PD/LBD.

### Cognitive deficits

Clinical studies relevant to this section are summarised in table 3.

**Table 3:** Clinical studies of GLP-1RAs for cognitive deficit

	Population	Exposure	Comparator	Follow-up	Outcomes
Pooled and combined analyses					
Luan *et al*^94^	7732 adults, T2DM (3 RCTs, 2 observational studies)	Dulaglutide, exenatide, liraglutide	Pretreatment baseline	3 months to 5 years	No overall improvements in cognitive function (MMSE, MoCA) with GLP-1RAs. Cognitive benefits in patients <65 years or without cardiocerebrovascular history.
Tian *et al*^72^	149 560 adults, T2DM (1 RCT, 3 observational studies)	GLP-1RAs	Non-users of GLP-1RA	4–7.2 years	GLP-1RAs (and SGLT-2is and thiazolidinediones) were significantly superior to other antidiabetics in reducing the risk of cognitive impairment.

Clinical trials					
Cheng *et al*^33^	36 adults, T2DM	Liraglutide	Acarbose, dapagliflozin	4 months	Liraglutide improved odour-induced hippocampal activation on fMRI and delayed memory, attention and executive function.
Cukierman-Yaffe *et al*^92^	9901 adults, T2DM; CV risk factors	Dulaglutide	Placebo	5.5 years	Reduced risk of substantive cognitive impairment adjusted for baseline scores (MoCA, DSST).
Dei Cas *et al*^93^	32 older adults, mild CI	Exenatide	No active comparator	8 months	No significant change in cognitive performance (ADAS-Cog-11). Potential benefits in female participants.
Li *et al*^34^	50 adults, T2DM	Liraglutide	No active comparator	3 months	Improved MMSE scores with liraglutide (independent of metabolic changes), associated with DLPFC and OFC activation on fNIRS.
Perna *et al*^89^	39 older adults, T2DM	Incretins (DPP-4i), liraglutide	SGLT-2i	1 year	No significant changes in cognitive performance (Verbal Fluency Test, Babcock Story Recall Test, Attentive Matrices Test).
Vadini *et al*^90^	40 adults, obesity, new T2DM or pre-diabetes	Liraglutide	Lifestyle intervention	3–6 months	Improved short-term memory and memory composite z-score with liraglutide (despite comparable weight loss and glycaemic control).
Wang *et al*^40^	60 older adults, T2DM, poststroke mild CI	Liraglutide	Sitagliptin	6 months	No improvements in MoCA and MMSE scores and in plasma markers of inflammation and Aβ aggregation with liraglutide.
Zhang *et al*^91^	19 adults, T2DM, with or without obesity	Exenatide, liraglutide	No active comparator	3 months	GLP-1RAs improved MoCA scores and odour-induced hippocampal activation on fMRI in subjects with obesity.

Observational studies					
Battini *et al*^87^	36 115 older adults, T2DM	Exenatide, liraglutide	DPP-4is	3 years	Lower risk of hospitalisation due to major CI in those treated with GLP-1RAs compared with DPP-4i. No significant effects for mild CI.
De Giorgi *et al*^57^	38 412–46 772 adults, T2DM	Semaglutide	Empagliflozin, glipizide, sitagliptin	1–2 years	Reduced risk of cognitive deficit with semaglutide compared with sitagliptin and glipizide (propensity score-matched cohorts).
Eren-Yazicioglu *et al*^86^	43 adults, T2DM and obesity	Exenatide	No active comparator	3 months	No significant differences in cognitive measures (Cognitive Failures Questionnaire and Letter-N-Back task).
Longo *et al*^51^	154 older adults, T2DM	Dulaglutide, liraglutide	No active comparator	>1 year	Higher MoCA and MMSE scores in patients treated with GLP-1RAs, associated with higher circulating levels of EPCs.

ADAS-Cog-11, Alzheimer’s Disease Assessment Scale-Cognitive; CI, cognitive impairment; CV, cardiovascular; DLPFC, dorsolateral prefrontal cortex; DPP-4i, dipeptidyl peptidase-4 inhibitor; DSST, Digit Symbol Substitution Test; EPCs, endothelial progenitor cells; fMRI, functional MRI; fNIRS, functional near-infrared spectroscopy; GLP-1RA, glucagon-like peptide-1 receptor agonist; MMSE, Mini-Mental State Examination; MoCA, Montreal Cognitive Assessment; OFC, orbitofrontal cortex; RCT, randomised controlled trial; SGLT-2i, sodium-glucose co-transporter-2 inhibitor; T2DM, type 2 diabetes mellitus.

Numerous studies investigated the effects of GLP-1RAs on more general definitions of cognitive impairment, especially in people with T2DM.^85^ There is also a growing literature^17^ around GLP-1RA use to improve cognition in the context of mental illness, especially depression and psychosis, which we do not cover in this review.

#### Observational studies

Observational evidence is scarcer in this area—reflecting the lack of granular information about cognitive scores in routinely collected data.

A cross-sectional analysis assessed 43 patients with T2DM and obesity in Turkey: 23 treated with exenatide and 20 without, finding no differences in self-reported and laboratory-based cognitive measures (all p>0.05, effect sizes not reported).^86^ A similarly designed study of 154 elderly Italian patients with T2DM conducted between 2018 and 2020 showed that GLP-1RAs plus metformin, compared with metformin alone, were associated with better Montreal Cognitive Assessment (MoCA) and Mini-Mental State Examination (MMSE) scores (all p<0.001, effect sizes not reported).^51^

Recently, a robust investigation of Danish registers has assessed the risk of hospitalisation due to either minor or major cognitive impairment at 3 years associated with DPP-4i against GLP-1RAs in older adults with T2DM: only major, but not minor, cognitive impairment-related hospitalisation was significantly higher in DPP-4i users (HR 1.58; 95% CI 1.22, 2.06).^87^ Similarly, our pharmacoepidemiological study of TriNetX US Collaborative Network EHRs^57^ showed lower hazards of cognitive deficits (defined as a composite outcome of various International Classification of Diseases-10th Revision codes reflecting cognitive impairment) within the first year since starting the GLP-1RA semaglutide compared with the DPP-4i sitagliptin (n=46 772; HR 0.72; 95% CI 0.64, 0.80) and the sulfonylurea glipizide (n=38 412; HR 0.72; 95% CI 0.63, 0.81), but not the SGLT-2i empagliflozin (which is known to also have some neuroprotective effects).^88^

These results are reassuring for patients with T2DM taking GLP-1RAs. Whether this premise holds true for other indications, such as in people with diabetes, remains to be determined.

#### Clinical trials

There are many clinical trials, although generally small, that examined cognitive performance in people treated with GLP-1RAs for T2DM. An early Italian trial randomised 39 adults with T2DM above 65 years old to either incretin (ie, the GLP-1RA liraglutide or several DPP-4is) or SGLT-2i therapy in addition to metformin and found no differences across cognitive tasks (all p>0.05, effect sizes not reported) at 1 year.^89^ In another similar trial in Italy including 40 metformin-treated individuals with pre-diabetes or newly diagnosed T2DM and obesity, liraglutide administration led to better memory scores compared with lifestyle counselling intervention (all p<0.05, effect sizes not reported) at variable study endpoints.^90^ Another short-term (12 weeks) trial of liraglutide compared with other antidiabetics in 50 Chinese patients with T2DM showed better scores in all cognitive tests including the MMSE, and especially in memory and attention domains (all p<0.05, effect sizes not reported)—interestingly, these scores correlated with increased activation of relevant brain regions (ie, DLPFC and OFC) but not with changes in metabolic parameters.^34^ Remarkably, similar findings were reported in two further Chinese trials with identical design at 12 weeks^91^ and for liraglutide only at 16 weeks.^33^ To date, the largest clinical trial (i.e., Researching Cardiovascular Events with a Weekly Incretin in Diabetes,REWIND) that provides useful data for cognition was conducted between 2011 and 2013 and randomly assigned 9901 participants with T2DM to either dulaglutide or placebo, showing a substantial reduction in cognitive impairment as measured on the MoCA in the intervention group (HR 0.86; 95% CI 0.79, 0.95) over a median of 5.5 years.^92^

Two trials involved people with pre-existing cognitive impairment. One conducted in China compared 30 people on the GLP-1RA liraglutide against 30 people on the DPP-4i sitagliptin—all participants had background T2DM and poststroke MCI.^40^ People assigned to sitagliptin had better MMSE and MoCA as well as lower Aβ and inflammatory markers (all p<0.001, effect sizes not reported) at 6 months, in contrast with most previous evidence showing that GLP-1RAs led to better cognitive outcomes than DPP-4i. Another trial in Italy compared exenatide with no treatment (ie, not placebo controlled) over 8 months in 32 people with a diagnosis of MCI and found no difference between groups (all p>0.05, effect sizes not reported) on the Alzheimer’s Disease Assessment Scale-Cognitive 11 scale, though a post hoc analysis showed a potentially beneficial interaction for female participants.^93^

At present, there is insufficient evidence to draw any firm conclusions from these trials—although the positive findings from the far larger REWIND trial^92^ for dulaglutide appear more promising and warrant further investigation.

#### Pooled and combined analyses

Only five^33 34 40 91 92^ of the clinical trials above, all of which had shown a significantly positive effect of various GLP-1RAs on cognition, were comprised in a meta-analysis including 7732 individuals with T2DM, which however unexpectedly did not observe a benefit on cognitive scores (SMD 0.33; 95% CI −0.03, 0.69) for these drugs.^94^ Two additional analyses already discussed above^71 72^ also assessed cognitive outcomes and reported that GLP-1RAs were better than other antidiabetics, but not SGLT-2i, in terms of cognitive scores. The same *caveats* discussed earlier regarding the interpretation of these studies still apply.

## Challenges and perspectives for research and clinical practice

In this review, we present and integrate evidence from mechanistic studies, including in humans, and clinical investigations about the putative neuroprotective effects of GLP-1RAs and their prospective use for the care of people with major neurocognitive disorders. As expected from such novel and promising research avenue, significant heterogeneity across several study designs, their methodological robustness and duration, target populations, individual drugs and dosages, as well as outcome measures, does not allow us to draw firm conclusions and ultimately limits our current interpretation of findings and application to routine practice. Certainly, the use of these medications, including off-licensed medical prescribing, is becoming exceptionally widespread as their indications expand beyond T2DM, weight loss and indeed the original predictions of the manufacturers,^95^ to an extent that has made people wonder whether GLP-1RAs may indeed be ‘good for everything’.^96 97^ To investigate their use in cognitive disorders, a number of important considerations need to be made, including brain penetrance; biomarkers; disease stage-based indication; non-specific effects on brain health; adverse events and other unwanted effects; long-term data; and cost and availability of treatment.

### Brain penetrance

The ability of GLP-1RAs to enter the CNS is debated, and we refer readers to ref 9 17 98 99 for a more extensive discussion of the topic. In short, robust studies in rodents demonstrate that all GLP-1RAs may cross the BBB but at largely variable rates and speed, seemingly higher for older agents (eg, exenatide, lixisenatide) and lower for newer ones (eg, semaglutide, tirzepatide).^100 101^ Even the larger and/or more electrically charged compounds, such as semaglutide, could still reach the brain via circumventricular sites and active transporters.^102 103^ Other mechanisms, such as brain capillary binding or sequestration, have been understudied.^99^ Interactions between the endogenous GLP-1 system, both peripheral and central, and how these may be perturbed by exogenous GLP-1RAs are unclear.^104 105^

Translating these findings in humans is complex. Based on the integration of current preclinical and clinical studies, some have argued that dulaglutide may be particularly penetrant.^106^ In people with T2DM, baseline glycaemic levels modulate GLP-1RA access to brain structures.^107^ Conversely, there is evidence of ‘leaky BBB’ in patients with neuropsychiatric diseases, including neurodegenerative disorders,^108^ which may facilitate CNS. Finally, it remains possible that some cognitive and behavioural effects of GLP-1RAs are mediated by their peripheral actions^9 17^—for instance, via the gut-brain axis and microbiome.^8 109^ In an attempt to improve brain penetrance while also limiting undesirable peripheral effects in people with cognitive disorders, intranasal formulations are being developed.^110^

Overall, we argue that whether and to what extent distinct GLP-1RAs penetrate the brain remains an unresolved issue, requiring more definitive studies in humans.

### Biomarkers

While biomarkers cannot be used in isolation to diagnose neurodegenerative conditions, they can guide clinical judgement on disease state and progression.^4^ There is a need for more robust biomarker references to improve the applicability of personalised treatments through understanding synergistic effects and overall efficacy (Tran, 2024 #160). Experimental medicine studies that incorporate the measurement of relevant biomarkers have served to validate measures of cognition and described the link between insulin and neurodegenerative disorders,^40^ and may provide further useful data to fill the translational gap from animal to human studies behind the putative neuroprotective mechanisms of action of GLP-1RAs (see figure 1 and the Mechanisms of neuroprotection section). Development of genetic, metabolic and neuroimaging-based biomarkers will be essential to the management of neurodegenerative disorders.^18^ GLP-1RAs have demonstrated effective anti-inflammatory properties and can improve insulin signalling, which supports a common biochemical pathway across neurodegenerative indications.^38^ Development of biomarkers that describe sensitivity of brain tissue to insulin may provide clinical guidance to help determine which individuals would respond to GLP-1RAs or combination therapies, which could in turn improve adherence and overall efficacy of intervention.^22^ In addition, sensitive biomarkers of neuroinflammation can advance targeted drug development in this area by allowing the demonstration of target engagement.

### Disease stage-based indication

Ongoing studies aim to bring clarity on whether there is an opportune stage to provide GLP-1RAs to those at risk or living with dementia.^68^ Offering adjunct therapies to GLP-1RA use in advanced stages of dementia, such as cholinesterase inhibitors and monoclonal antibodies, raises the possibility of synergistic effects, such as neuroinflammation and insulin signalling modulation potentiating the effects of amyloid clearance.^4^ [Kopp *et al* 2024] Treatment as either monotherapy or adjunct therapy would require future studies to determine the optimal disease stage for intervention.

### Non-specific effects on brain health

Besides the several neuroprotective mechanisms putatively assigned to GLP-1RAs (see figure 1 and the Mechanisms of neuroprotection section), their largest contribution to a healthy cognition might be due to non-brain-specific benefits on other health domains.^17^ We have already mentioned the connections between diabetes, obesity and major neurocognitive disorders, where GLP-1RAs may act fruitfully by breaking such instrumental links. Another example entails the established cardioprotective activity of GLP-1RAs,^111^ perhaps together with other helpful actions on brain perfusion as seen in the Cerebrovascular system and BBB dynamics section,^50^ which may have direct relevance to controlling risk factors such as hypertension and atherosclerosis,^112^ hence to the prevention of non-fatal stroke events and ultimately VaD.^13^ GLP-1RAs are also being investigated for the treatment of addiction, with preliminary evidence suggesting that they might be useful for reducing smoking^57 113^ and alcohol misuse^114^—important risk factors for AD, alcohol-related dementia and other cognitive impairments.^115^ The development of novel GLP-1RAs could therefore be integrated with lifestyle interventions directly impacting key determinants of health^116^ to improve brain health and healthy ageing.^117^

### Adverse events and other unwanted effects

Studies thus far have broadly reported that GLP-1RAs display a benign safety profile,^6^ including in terms of adverse neuropsychiatric outcomes.^17^ Gastrointestinal symptoms, including nausea, bloating and sometimes vomiting, are the most common side effects, generally occurring during the first few weeks of taking the medications and subsiding with prolonged administrations.^6^ Some controversy remains regarding the relationship between these drugs and suicidality.^118^ Within the context of CNS disorders, pharmaceutical companies and researchers should also consider the development of GLP-1 agents that do *not* cause weight loss, especially of lean muscle mass—a peripheral effect that would be undesirable in frail older adults.

### Long-term data

Within the field, there is a lack of longitudinal data to assess the long-term efficacy and safety risks of GLP-1RAs in an ageing population with preclinical or syndromic neurodegeneration associated with frailty. There is evidence that insulin receptor desensitisation may result in limited long-term use. However, animal studies have shown that GLP-1 agents with adjunctive therapies or an atypical dosing routine could mitigate receptors’ desensitisation.^10^ Several studies have reported a few cases of pancreatic cancer and gastrointestinal complications, which require further evaluation to consider whether this is an associated risk. [Marx *et al*; ^82^] Further research should plan to evaluate risks of long-term use with considerations to age, comorbidities and impact of fluctuating mental capacity that increases the risk of self-harm.^118^

### Cost and availability of treatment

Elevated costs associated with GLP-1RAs may limit accessibility, both in high-income and especially in low- and middle-income countries. Moreover, since the introduction of semaglutide, severe drug shortages have also been common. Efforts to reduce production costs (eg, cheaper injection devices, non-injectable formulations) and to explore generic formulations could improve affordability and expand global access to these therapies.^119^ Cost-effectiveness analyses are essential to justify the widespread use of GLP-1RAs. Notably, only one study has demonstrated the cost-effectiveness of add-on exenatide in PD,^120^ and none for other molecules or disorders. Further engagement between researchers and policymakers is vital to inform health policy reforms that support reimbursement and facilitate broader adoption of innovative therapies with substantial impact on public health such as GLP-1RAs.

## Conclusions

In conclusion, emerging GLP-1RAs may be critical to address unmet needs for the growing numbers of people suffering from major neurocognitive disorders.^121^ These medications might have multifaceted neuroprotective effects involving brain energy homeostasis, neurogenesis, synaptic functioning, neuroinflammation and other cellular stress responses, pathological protein aggregates, proteostasis, cerebrovascular system and BBB dynamics, which might prove instrumental in tackling the complex pathophysiology behind neurodegenerative processes. Notwithstanding some inconsistencies and translational gaps, existing clinical studies in dementia, including AD, PD/LBD and other cognitive deficits, are encouraging and perhaps even suggestive of GLP-1RA disease-modifying properties. Perspectives for further advances via the concerted effort of industry, academic institutions and other stakeholders are likely within the prevailing research landscape. Several challenges remain that need addressing through rigorous studies to pave the way for delivering GLP-1RAs into routine clinical practice for the benefit of patients with cognitive disorders.
